# Butyrate enhances recovery of chemotherapy-induced oral mucositis by enhancing tight junction protein expression and inhibiting inflammatory responses

**DOI:** 10.4317/medoral.27080

**Published:** 2025-08-16

**Authors:** Jing-jing Zeng, Zeng-lin Tan, Ke Zhang, Qian-nian Han, Shi-ning Ou, Ze-kai Wang, Jian-wei Liu, Fang-yan Wang, Ying-peng Huang, Lin-feng Wu

**Affiliations:** 1 Cixi Biomedical Research Institute, Wenzhou Medical University, Zhejiang, China; 2 Oncology and Hematology, Wenzhou Hospital of Integrated Traditional Chinese and Western Medicine, Wenzhou, China; 3 Department of Orthopaedics, The First Affiliated Hospital of Wenzhou Medical University, Wenzhou, China; 4 Department of Pathophysiology, School of Basic Medical Sciences, Wenzhou Medical University, Wenzhou, China; 5 Department of General Surgery, Second Affiliated Hospital of Wenzhou Medical University, Wenzhou, China

## Abstract

**Background:**

Chemotherapy-induced oral mucositis (OM) is a common complication of cancer treatment that significantly impacts patients' quality of life. Butyrate, a short-chain fatty acid, has been shown to inhibit inflammation and mitigate intestinal mucosal damage. How, its effect in treating OM remains unclear.

**Material and Methods:**

OM model in mice pretreated with 5-FU solution was established by injecting 20% acetic acid into oral mucosa. Sodium butyrate was given for treatment. H&E staining was used to observe histopathological changes, and qRT-PCR to assess inflammatory factor level changes in ulcer tissues after treatment. Also, qRT-PCR and immunofluorescence staining were used to evaluate the expression and distribution of tight junction protein in ulcer tissues.

**Results:**

Sodium butyrate treatment improved the weight loss in mice caused by OM and promoted the repair of oral mucosa in a time-dependent manner. In addition, sodium butyrate significantly inhibited the mRNA levels of inflammatory factors such as tumor necrosis factor-α (TNF-α), interleukin-1β (IL-1β), interleukin-6 (IL-6), and interleukin-18 (IL-18) in the ulcer tissue. Moreover, sodium butyrate promoted the mRNA and protein expressions of tight junction protein-1 (ZO-1) and Claudin-1 in the epithelial cells of the ulcer tissue.

**Conclusions:**

Butyrate promotes OM healing by reducing inflammation and increasing the expression of tight junction proteins in ulcer tissues.

** Key words:**Butyrate, chemotherapy, oral mucositis, inflammation, tight junction proteins.

## Introduction

Chemotherapy (CT) is a fundamental anti-tumor treatment that employs cytotoxic chemicals to eliminate tumor cells, which are characterized by the high proliferation rates. However, certain normal tissue cells with robust regenerative capability are inevitably affected by these chemotherapeutic agents due to the lack of precise targeting, such as oral mucosa (1). Oral mucositis (OM), characterized by oral mucosal erythema and ulcers, is the most common complication among chemotherapy patients (2). Dysphagia, significant reductions in food intake, secondary oral infections, and even systemic infections resulting from the mucosal lesions and severe pain associated with OM can lead to decreased compliance with chemotherapy and negatively impact patient survival and quality of life (2-4). Currently, treatment options for OM are limited, but innovative therapies based on its underlying pathogenesis show promising clinical applications. The release of inflammatory factors, such as tumor necrosis factor, interleukin (IL)-1, and IL-6, alongside decreased expression of anti-inflammatory factors like IL-10 and transforming growth factor (TGF) β, is closely associated with the pathogenesis of OM (5,6).

Butyrate, a short-chain fatty acid primarily produced by intestinal flora (7), serves as an important energy source and possesses anti-inflammatory properties that protect intestinal barrier function (8,9). Studies have shown that butyrate can downregulate the expression of pro-inflammatory factors (such as IL-6, IL-8, and TNF-α) to alleviate colitis (10). Dietary supplementation with butyrate can reduce inflammation of the intestinal mucosa caused by cytarabine in mice (11); moreover, butyrate effectively inhibits inflammation and reduces intestinal mucosal injury in mice induced by 5-fluorouracil (5-FU)(12). In addition to its anticipated role in suppressing inflammation, butyrate is crucial for maintaining epithelial barrier function. It can upregulate the expression of mucin genes in human intestinal mucosal epithelial cells and enhance mucosal barrier function (13,14). Butyrate also regulates the expression of tight junction proteins through lipoxygenase activation, thereby reducing mucosal permeability (15). Based on these findings, we hypothesized that butyrate, by reducing inflammation and modulating protein expression, may improve oral mucosal inflammation and barrier damage caused by chemotherapy, warranting further exploration of its protective mechanisms on the oral mucosa.

In this study, we demonstrated that butyrate promotes mucosal repair by reducing the levels of inflammatory factors, including IL-6, IL-1β, IL-18, and TNF-α, while enhancing the expression of tight junction proteins ZO-1 and Claudin-1.

## Material and Methods

- Experimental animals and reagents

All animal experiments were conducted with the approval of the Animal Experimental Ethical Committee of Wenzhou Medical University. 40 ICR male mice, weighing approximately 25-30g, were provided by Zhejiang Muke Biological Co., LTD. ICR mice were placed in a room with a constant temperature (22±1 ℃) for 12 hours of light/dark cycle, and fed with standard pellet feed and water AD libitum. Acetic acid (Shanghai chemical reagent co., LTD); Chloral hydrate; Sodium butyrate (Aladdin ®); 5 - FU (Aladdin ®).

- Oral ulcer in mice induced 5 - FU model

40 ICR mice were randomly divided into two groups: treatment group (*n*=20) and model group (*n*=20), Separate cage feeding. In the first 5, 3, 1 [-5, -3, -1] days of modeling, Every mice celiac injection of 4 mg/ml 5 - FU (made from the physiological saline), dose of 40 mg/kg. Subsequently, under 2% chloral hydrate anesthesia on day 0, 15μl 20% acetic acid was injected into the left cheek with a 31-G needle to induce mucosal ulceration. On the third day after modeling, the ulcer was basically formed in the mice. In the following days, the ulcer changes, inflammation and congestion were observed, and the healing condition was observed by photographing and the healing time was recorded.

- Histopathological analysis

On the 8th and 12th day of oral ulcer formation, 6 ICR mice were randomly selected from each group. After anesthesia with 2% chloral hydrate, the ulcer tissues were cut off, fixed with 4% paraformaldehyde for 24 hours, dehydrated in ethanol gradient, embedded in paraffin, and serially sectioned (4um) for HE staining.

- Tissue immunofluorescence

The expressions of tight junction proteins Claudin-1 and ZO-1 in ulcer tissues were detected. After 24h of fixation with 4% paraformaldehyde, the plates were embedded in paraffin and sectioned. After drying at 60° for 1h, sections were dewaxed and rehydrated, repaired with sodium citrate antigen repair solution (PH=6), permeabilized with 0.5% Triton-100 for 5min, blocked with 1% BSA for 30min, and incubated with primary antibodies against Claudin-1 (Proteintech, 28674-1, 1:400) and against ZO-1 (Proteintech, 66452-1, 1:400) at 4°C overnight in a wet box. The sections were incubated with the secondary antibody at room tempera ture for 1 h after washing with PBS.

The slides were sealed with anti-quench sealing agent containing DAPI and observed under fluorescence microscopy.

- Real-time fluorescence quantitative PCR

The excised oral ulcer tissue was put into a 1.5ml enzyme-free centrifuge tube, 1ml Trizol (Yamei, Shanghai, China) was added, the tissue was fully ground, left at room temperature for 5min, 200ul chloroform was added, mixed, and centrifuged at 12000xg 4℃ for 15min in a pre-cooled centrifuge. 400ul of upper aqueous phase (RNA) was sucked and transferred to a new 1.5ml enzyme-free centrifuge tube, and the equal volume of isopropanol was added, mixed, left at room temperature for 10min, centrifuged at 12000xg 4℃ for 10min, and RNA precipitation was seen at the bottom of the discarded supernatant, then 75% ethanol prepared with DEPC water was added to dissolve, and the supernatant was centrifuged to remove. The RNA precipitate was allowed to dry naturally at room temperature, RNA-free water was added to dissolve the RNA, and the concentration was determined. The isolated RNA was reverse-transcribed to cDNA using a kit (Vazyme, Nanjing, China), and real-time quantitative PCR amplification was performed on a PCR instrument. mRNA expression levels were analyzed by the 2-ΔΔCt method using GAPDH as an internal control.

- Statistical analysis

GraphPad Prism 9.0.2 was used for statistical analysis. The error lines in the Figures represent SEM. * *P* < 0.05, ** *P* < 0.01, * * * *P* < 0.001, **** *P* < 0.0001. Significance (*P* value) was calculated using ordinary one-way ANOVA one-way analysis of variance (ANOVA) for differences between groups (data followed normal distribution) or Kruskal-Wallis test non-parametric test (data did not follow normal distribution). Normality was tested using the Shapiro-Wilk test.

## Results

- 5-FU doses impact mouse survival and weight

**Figure 1:**
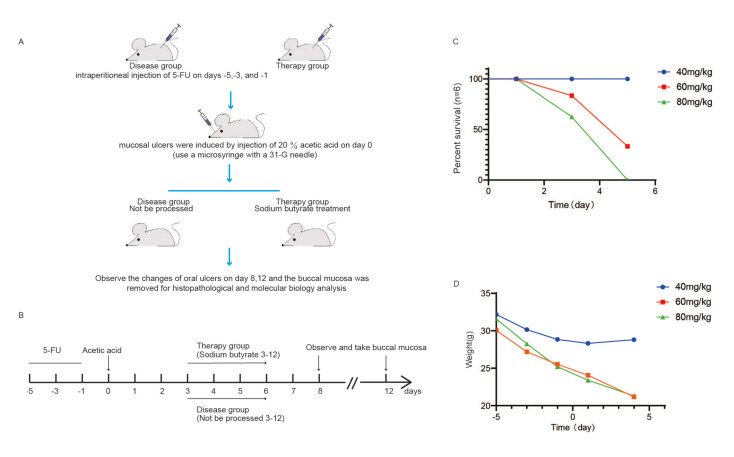
Experiment process and 5 - FU dosage determined. (A-B) Flow chart of the experiment (*n*=6/group). The survival rate (C) and body weight (D) of the mice under different doses (*n*=6/group).

The experimental process is illustrated in Fig. 1. A mouse ulcer model was established using different dose of 5-fluorouracil and 20% acetic acid injection. Administration of 40 mg/kg 5-FU did not affect mouse survival, whereas doses of 60 and 80 mg/kg significantly impacted survival (Fig. 1). Administration of 40 mg/kg 5-FU on day 0 had no significant effect on mouse body weight, whereas doses of 60 and 80 mg/kg resulted in weight loss (Fig. 1).

- Butyrate alleviates oral ulcers and enhances appetite

**Figure 2:**
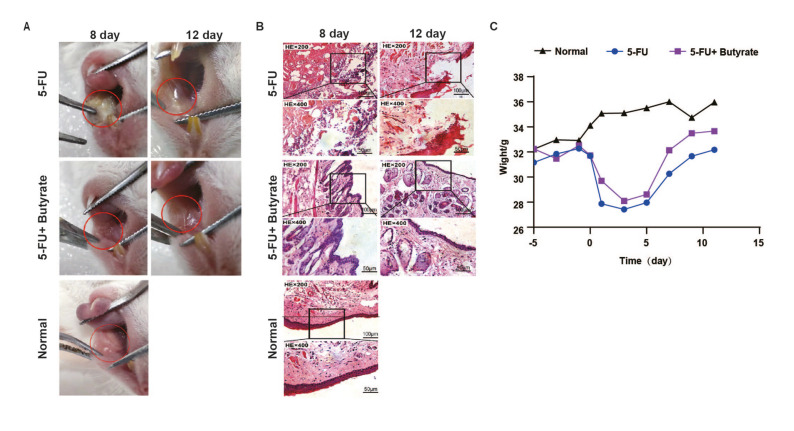
Butyrate alleviated oral ulcers in mice. (A) Mouse oral mucosa ulcer group size. (B) Representative H&E staining images of the oral mucosa. (C) The image shows the weight of mice (*n*=6/group).

Butyrate treatment significantly reduced oral mucosal lesions in OM mice and restored the integrity of the mucosa (Fig. 2). With prolonged butyrate intervention, the appetite of the mice significantly improved, and their body weight showed a remarkable recovery compared to the oral ulcer group (Fig. 2).

- Butyrate restores tight junction protein expression

**Figure 3:**
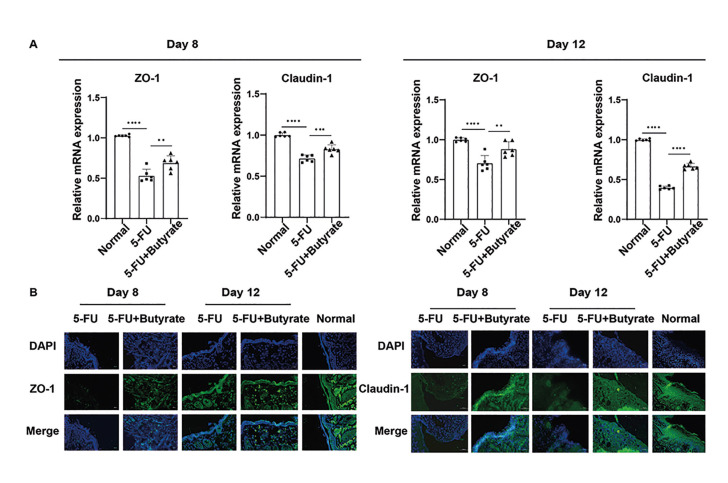
Butyrate promoted the expression of tight junction proteins in the mucosal epithelium. (A) Transcriptional levels of tight junction protein in mouse mucosa (*n*=6/group). (B) Immunofluorescence staining of paraffin sections of Claudin-1 and ZO-1 proteins(n=6/group).

Transcription levels of tight junction proteins ZO-1 and Claudin-1 were significantly lower in the oral ulcer group compared to the normal group. Following 8 and 12 days of butyrate intervention, transcription levels of ZO-1 and Claudin-1 were significantly upregulated in butyrate-treated mice when compared to the oral ulcer group (Fig. 3). Additionally, immunofluorescence results demonstrated that protein levels of ZO-1 and Claudin-1 were significantly elevated in butyrate-treated mice relative to those with oral ulcers (Fig. 3).

- Butyrate reduces mucosal inflammatory factor levels

**Figure 4:**
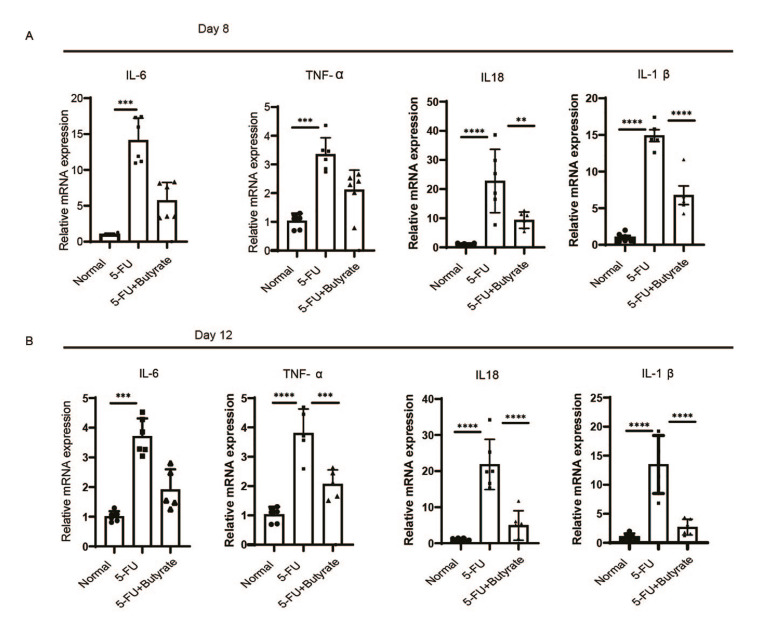
Butyrate relieve inflammation of the mucous membrane. Transcript levels of inflammatory factors in the oral mucosal. (A) the eighth day (*n*=6/group). (B) The twelfth day (*n*=6/group).

The levels of inflammatory cytokines IL-6, TNF-α, IL-18, and IL-1β in the oral ulcer group were significantly higher than in the normal group. After 8 days of butyrate intervention, the inflammatory factors IL-1β and IL-18 were significantly downregulated in the butyrate-treated group (Fig. 4). Furthermore, by day 12 of the intervention, the levels of TNF-α, IL-18, and IL-1β were also significantly reduced in the butyrate group (Fig. 4).

## Discussion

OM is a common side effect of chemotherapy, with an incidence reported at 40%. OM induced by anticancer drug therapy manifests as erythema, edema, or ulceration, which can range from mild burning sensations to extensive, painful ulcers, presenting more severely than general stomatitis. Symptoms associated with OM include difficulties in eating, communication, and sleep, along with significant pain, all of which can diminish the patient's quality of life. Severe cases of OM can lead to loss of consciousness and necessitate treatment cessation. Therefore, effective prevention or treatment of OM is crucial for improving patients' quality of life and reducing the likelihood of treatment interruption. Currently, various therapeutic approaches—including oral cryotherapy, oral hygiene practices, corticosteroids, and antiseptic mouthwashes—have been implemented (16). However, these strategies often fall short in alleviating or preventing OM (17), highlighting the need for new treatments targeting its pathogenesis. This study investigates the therapeutic potential of butyrate in the management of OM.

In this study, we established an OM model based on previous research by administering an intraperitoneal injection of 5-FU solution (8 mg/ml) combined with 15 µl of 20% acetic acid on the left cheek of mice to induce mucosal ulceration (18,19). The dosage of 5-FU was controlled at 40 mg/kg per mouse, a level that did not significantly affect the short-term survival rate and body weight of the mice. On day three post-OM model establishment with 5-FU, the ulcer area in the mice reached its maximum size. 5-FU is an antimetabolite commonly utilized as a standalone therapy or as part of combination regimens for various cancers, including colorectal, breast, respiratory, and digestive tract malignancies (20). Recent animal studies have demonstrated that 5-FU induced oral mucosal atrophy, accompanied by upregulation of pro-inflammatory mediators such as TNF-α, IL-1β, and IL-6, and led to impairment of epithelial cell barrier integrity (21). Our findings corroborated these observations, revealing significant increases in inflammatory factors like IL-1β, IL-6, IL-18, and TNF-α, as well as marked downregulation of tight junction proteins such as Claudin-1 and ZO-1 in the OM model mice compared to normal controls. These results suggest that inflammatory reactions and the destruction of epithelial barrier function occur within the ulcerative tissue.

Short-chain fatty acids (SCFAs), including acetate, propionate, and butyrate, are metabolites of dietary fiber that are metabolized by the gut microbiota and their effects on health have been extensively studied (22). Butyrate, a four-carbon SCFA, serves as a primary energy source for colonic epithelial cells and significantly influences their growth and differentiation (23). Additionally, butyrate plays a crucial role in modulating immune and inflammatory responses (24). In the context of inflammatory bowel disease, butyrate has been shown to inhibit neutrophil migration and the release of inflammatory mediators and chemokines, thereby reducing mucosal inflammation (25). Furthermore, butyrate enhances the expression of tight junction proteins such as ZO-1, occludin, and Claudin-1, thus improving intestinal barrier function (26). Given butyrate's properties in mitigating inflammation and enhancing epithelial barrier function, its potential application in treating OM is worth exploring. In our study, butyrate was applied daily to the ulcer site in the treatment group from day three of the OM model for a duration of nine days. Treatment resulted in a significant reduction in ulcer area compared to the untreated OM group. mRNA levels of IL-1β, IL-6, IL-18, and TNF-α in the ulcer tissue were significantly reduced following butyrate treatment, while the levels of tight junction proteins Claudin-1 and ZO-1 were significantly elevated. These data indicate that butyrate effectively inhibits inflammation and restores barrier function.

DNA damage and ROS simultaneously activate the nuclear factor kappa-light-chain-enhancer of activated B cells (NF-kB) transcription factor, leading to the up-regulation of more than 200 genes, many of which are associated with mucosal toxicity (27). and increased levels of pro-apoptotic factors such as BAX that promote epithelial cell apoptosis (28).A disruption to the mucosal barrier can result in an inflammatory response, characterised by an increase in the transcription levels of pro-inflammatory mediators, including TNF-α and IL-6. These mediators contain NF-κB binding sites in their promoters and enhancers, which play a crucial role in regulating the inflammatory cascade.This cascade of inflammations a potential consequence of the accumulation of reactive oxygen species in tissues (29,30).We hypothesized that butyrate may alleviate oxidative stress and epithelial cell apoptosis by repairing mucosal barrier function. However, this part of the work is being further refined.

In conclusion, our findings confirm that butyrate mitigates the 5-FU-induced OM by inhibiting inflammation and promoting the expression of tight junction proteins, Claudin-1 and ZO-1.
